# Pinocembrin–Lecithin Complex: Characterization, Solubilization, and Antioxidant Activities

**DOI:** 10.3390/biom8020041

**Published:** 2018-06-19

**Authors:** Xu Yang, Xin Wang, Xiao-Yu Chen, Hai-Yu Ji, Yan Zhang, An-Jun Liu

**Affiliations:** Key Laboratory of Food Nutrition and Safety, Ministry of Education, School of Food Engineering and Biotechnology, Tianjin University of Science and Technology, Tianjin 300457, China; wangxin392@163.com (X.W.); m15522377851@163.com (X.-Y.C.); jihaiyu1247@163.com (H.-Y.J.); cpzyyan@126.com (Y.Z.)

**Keywords:** pinocembrin–lecithin complex, solubility

## Abstract

Pinocembrin is a natural flavonoid compound which is capable of antioxidant, antibacterial, anti-inflammatory, and antineoplastic activities. The present study aimed to enhance the solubility and antioxidant activities of pinocembrin by complex formation with lecithin. The physicochemical characteristics of pinocembrin–lecithin complex were analyzed by ultraviolet (UV), fourier-transform infrared spectroscopy (FTIR), scanning electron microscopy (SEM), differential scanning calorimetry (DSC), and solubility assay, and the antioxidant activities of pinocembrin–lecithin complex were evaluated via radical scavenging capacities for 2,2′-diphenyl-1-picrylhydrazyl (DPPH), 2,2′-azino-bis(3-ethylbenzthiazoline-6-sulphonic acid) (ABTS), hydroxyl, and superoxide-anion. The results indicated that pinocembrin complex with lecithin could significantly improve the solubility of pinocembrin in water and *n*-octane, the pinocembrin–lecithin complex displayed no characteristic endothermic peak and the appearance of amorphous state, compared to the pinocembrin, and no new covalent bond was produced in the pinocembrin and lecithin compound. It was demonstrated that the antioxidant activities of pinocembrin were obviously enhanced by the complex with lecithin, and the scavenging capacities for hydroxyl radical, DPPH, superoxide-anion radical, and ABTS radical of pinocembrin–lecithin complex were 82.44 ± 2.21%, 40.07 ± 1.32%, 59.15 ± 0.86%, and 24.73 ± 1.04% at 1.0 mg/mL, respectively. It suggested that the pinocembrin–lecithin complex had a great potential application prospect in the healthcare industry and in clinical practice.

## 1. Introduction

Pinocembrin ((*S*)-5,7-dihydroxyflavanone) is a natural flavonoid compound that has been found in propolis, honey, and several plants, such as *Glycyrrhiza glabra*, ginger roots, and wild marjoram [1,2]. Apart from being extracted from natural sources, pinocembrin has also been successfully chemosynthesized or biosynthesized artificially [3,4]. It has been reported to exhibit a variety of pharmacological activities including antibacterial, anti-inflammatory, antioxidant, vasodilative, antiprotozoal, neuroprotective, and antiproliferative properties [5,6,7,8,9,10]. Furthermore, studies have revealed that pinocembrin could protect the brains of rats against acute cerebral ischemia by reducing the area of cerebral infarction, maintaining the structure of brain mitochondria, and alleviating blood–brain barrier injury [11,12,13,14]. Pinocembrin could be developed as a new drug to treat ischemic strokes, and now it is in a phase II clinical trial [15]. In addition, pinocembrin can be used against colon cancer because of its ability to trigger bax-dependent mitochondrial apoptosis [16]. Furthermore, pinocembrin could be utilized as a natural insect antifeedant and a new tool for protecting plants from harmful insects, especially in organic agriculture [17]. Therefore, pinocembrin is a great latent healthcare prospect which must be explored for its uses associated with the pharmaceutical sector. However, the application of pinocembrin in clinic practice, as a natural herbal medicine, is greatly limited by its low solubility and bioavailability [18,19].

Lecithin has a favorable surfactivity owing to a hydrophilic phosphate group and a hydrophobic fatty acid group. It is well known that lecithin is able to self-assemble in water in form of pile of bilayers (lamellar phase) which in proper conditions can lead to the formation of closed vesicles (liposomes) [20]. The natural active ingredient and lecithin compound has been proved to availably heighten the gastrointestinal tract absorption of the natural active ingredient, significantly improving its bioactivity and obviously reducing its toxicity and dosage [21,22]. Lecithin is widely employed to enhance the stability, solubility, and bioavailability of guests because of its low price and high production rate [23,24].

This study was undertaken with the objective to improve the solubility and antioxidant activities of pinocembrin by complex formation with lecithin. For this, the pinocembrin–lecithin complex was prepared and characterized. Its physical and chemical characteristics were investigated by the methods showed in Table 1. Additionally, the antioxidant activity of the pinocembrin–lecithin complex was evaluated via radical scavenging capacities for 2,2′-diphenyl-1-picrylhydrazyl (DPPH), 2,2′-azino-bis (3-ethylben-zothiazoline-6- sulphonic acid) (ABTS), hydroxyl, and superoxide-anion in comparison with pure pinocembrin.

## 2. Results

### 2.1. Solubility Analysis

As illustrated in Table 2, both the apparent solubility in water (265.00 μg/mL) and *n*-octane (165.24 μg/mL) of the pinocembrin complex with lecithin were the greatest in the three substances; being over four times and one and a half times higher than those of pinocembrin (48.33 and 65.24 μg/mL), respectively. These results suggested that the lecithin was likely to improve water solubility and lipid solubility of pinocembrin.

### 2.2. Ultraviolet and Infrared Analysis

**Figure 1:**
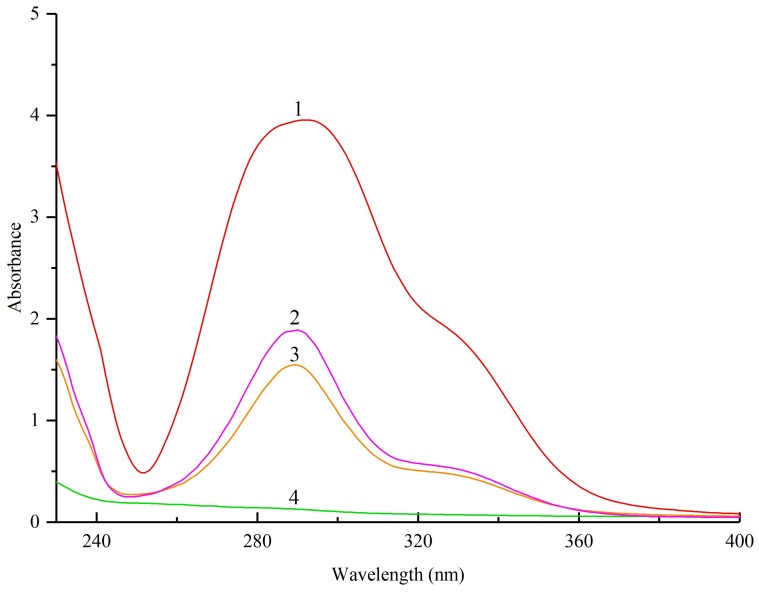
Ultraviolet (UV) spectra analysis of: (**1**) pinocembrin; (**2**) pinocembrin complex with lecithin; (**3**) physical mixture of pinocembrin and lecithin; (**4**) lecithin.

The ultraviolet (UV) spectra of pinocembrin, lecithin, their physical mixture, and the complex are shown in Figure 1. All of the pinocembrin, physical mixture, and the complex with lecithin displayed a maximum absorption at 290 nm with a similar elementary morphology, and the height of these absorption peaks was different for each, which suggested that the structure of the main chromophoric group of pinocembrin was not modified in the course of compound formation.

**Figure 2:**
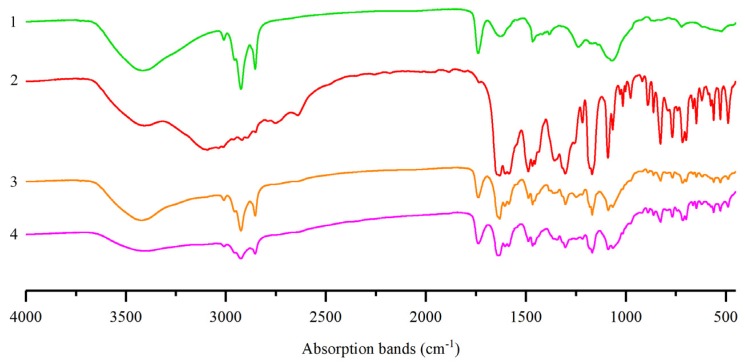
Infrared (IR) spectra analysis of: (**1**) lecithin; (**2**) pinocembrin; (**3**) physical mixture of pinocembrin and lecithin; (**4**) pinocembrin complex with lecithin.

To explore the possibility of any interaction between pinocembrin and lecithin, a fourier-transform infrared (FTIR) spectra analysis was performed. As shown in Figure 2, the FTIR spectrum of lecithin exhibited the conspicuous absorption bands at 2924 cm^−1^ and 1740 cm^−1^, which was the C–H stretching vibration and C=O stretching vibration, respectively. The FTIR spectra of the physical mixture and the complex manifested approximate superimposition of individual patterns of pinocembrin and lecithin. However, some small characteristic absorption peaks of the complex between 1500 cm^−1^ and 500 cm^−1^ were almost masked by the lecithin, which implied that some feeble physical interactions between pinocembrin and lecithin happened during the formation of the complex [21]. The main characteristic absorption peaks of pinocembrin and lecithin were also seen in the spectra of the pinocembrin–lecithin complex, and no novel absorption peak was found, revealing that no novel covalent bond was generated in the pinocembrin and lecithin compound.

### 2.3. Scanning Electron Microscopy Analysis

**Figure 3:**
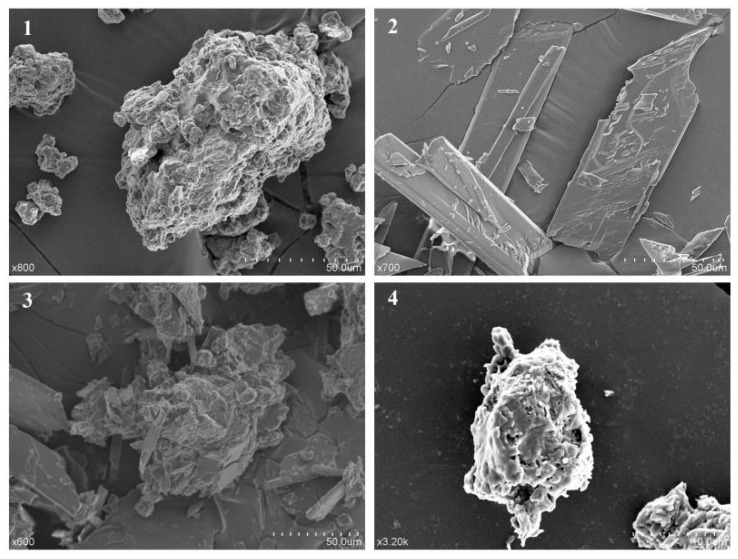
(**1**) Scanning electron microscopy (SEM) analysis of lecithin; (**2**) pinocembrin; (**3**) physical mixture of pinocembrin and lecithin; (**4**) pinocembrin complex with lecithin.

The apparent morphology of pinocembrin, lecithin, their physical mixture, and the complex measured by scanning electron microscopy (SEM) is shown in Figure 3. The lecithin presented an amorphous state and the pinocembrin exhibited an acicular crystal form. The SEM image of the physical mixture indicated that the acicular crystal form of pinocembrin was not vanished but rather was dispersed in the lecithin. The pinocembrin complex with lecithin displayed a similar amorphous form to the lecithin, which implied that the pinocembrin was embedded into the lecithin and behaved as in an amorphous state.

### 2.4. Differential Scanning Calorimetry Analysis

**Figure 4:**
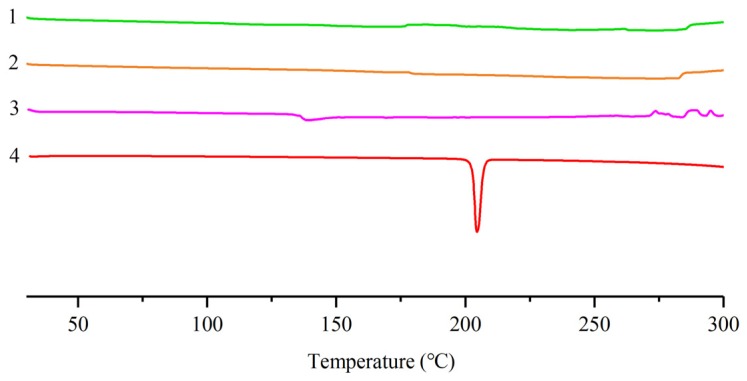
(**1**) Differential scanning calorimetry (DSC) curves of lecithin; (**2**) physical mixture of pinocembrin and lecithin; (**3**) pinocembrin complex with lecithin; (**4**) pinocembrin.

The differential scanning calorimetry (DSC) thermograms of pinocembrin, lecithin, their physical mixture, and the complex are exhibited in Figure 4. The endothermic peak of pinocembrin was at about 204 °C whereby the pinocembrin started to melt. Because the lecithin was a kind of amorphous substance, there were no constant fusion point and no distinct endothermic peak in the DSC curve of lecithin. The DSC thermograms among the lecithin, the physical mixture of pinocembrin and lecithin, and the pinocembrin–lecithin complex were so remarkably similar that the characteristic endothermic peak of pinocembrin disappeared. It was considered that the characteristic endothermic peak of pinocembrin had been masked by the lecithin and there was some interaction between them, such as the combination of hydrogen bonds or van der Waals force [22]. The results are well supported by previous studies, which were related to the lecithin complexes with phytoconstituents such as formononetin and polydatin [25,26].

### 2.5. Antioxidant Activities In Vitro

**Figure 5:**
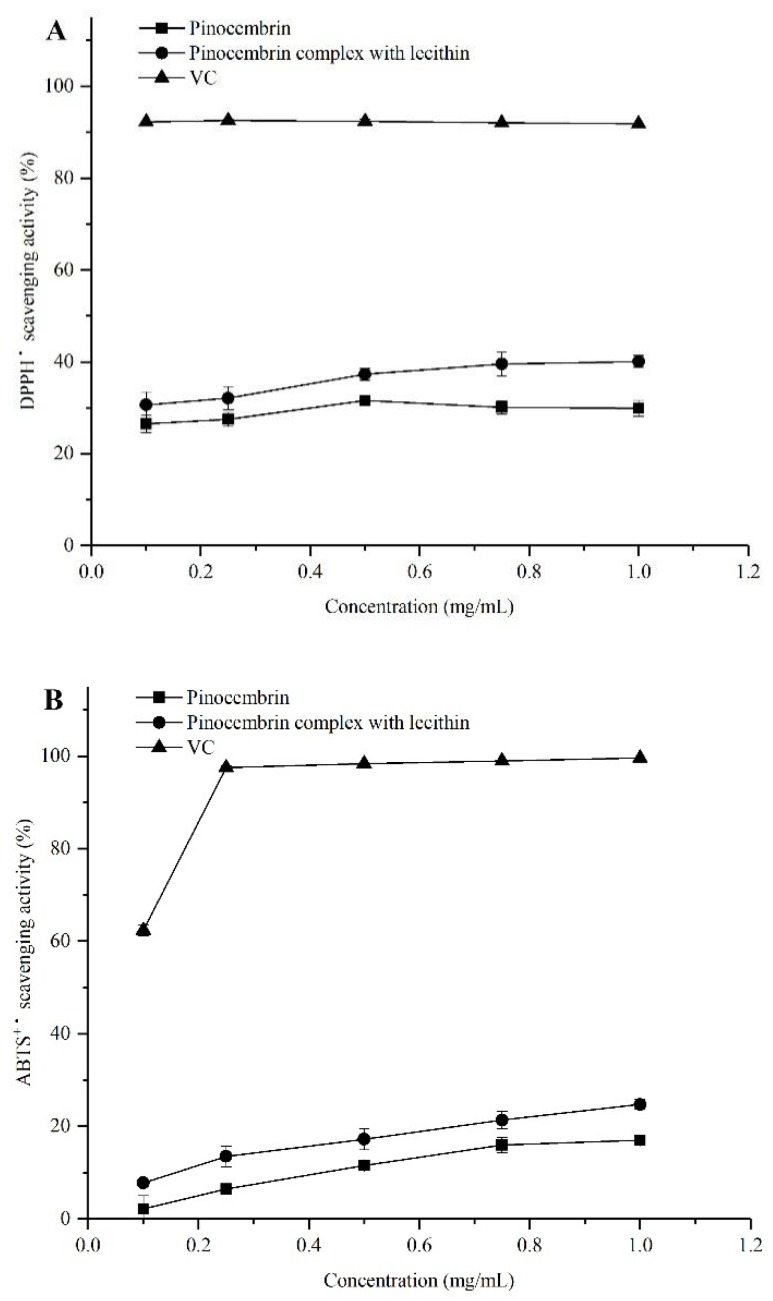

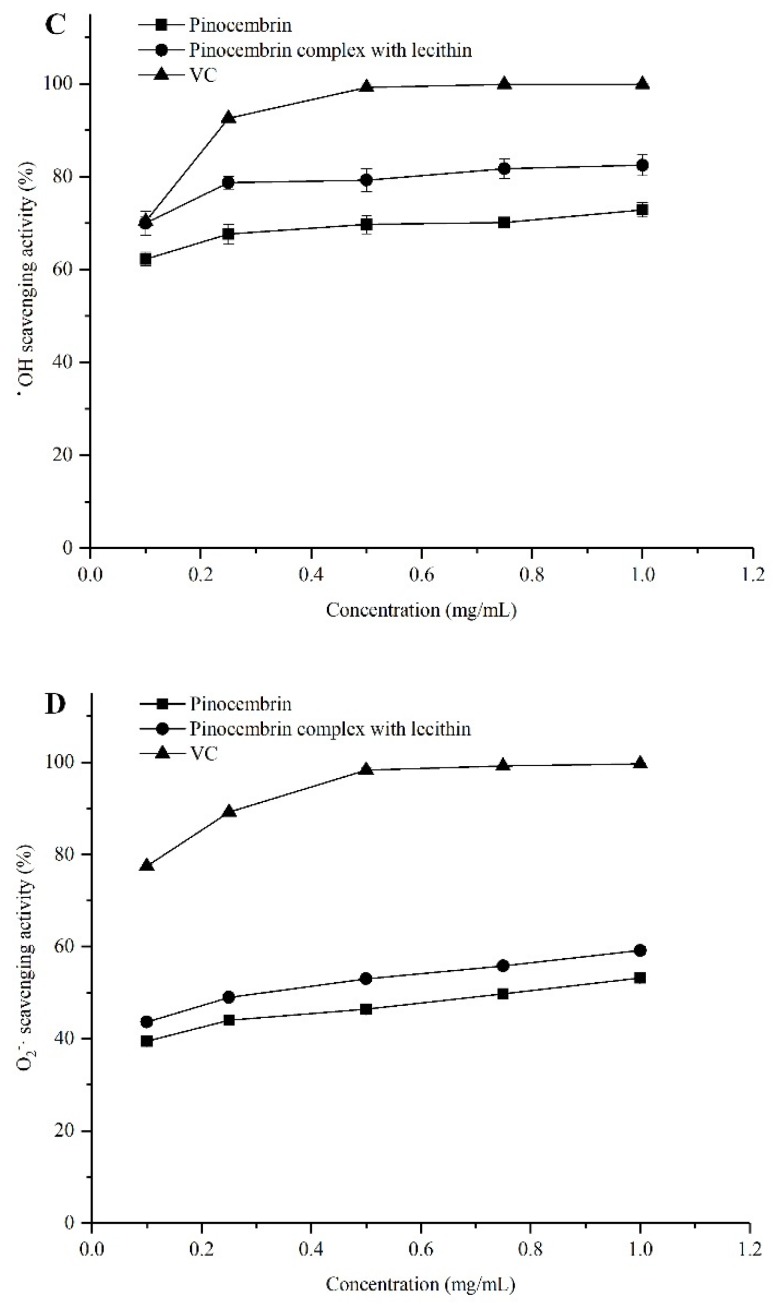
Antioxidant activities of pinocembrin and its complex. (**A**) 2,2′-diphenyl-1-picrylhydrazyl (DPPH) radical scavenging activity; (**B**) 2,2′-azino-bis(3-ethylbenzthiazoline-6-sulphonic acid) (ABTS) radical scavenging activity; (**C**) hydroxyl radical scavenging activity; (**D**) superoxide-anion scavenging activity.

The antioxidant capabilities of pinocembrin and the pinocembrin–lecithin complex were evaluated by a series of assays (radical scavenging capacity for DPPH, ABTS, hydroxyl, and superoxide-anion), because of the difference in the antioxidant action mechanisms [27]. The results are shown in Figure 5. In all the antioxidant experiments, the antioxidant capacities of pinocembrin and its complex were weaker than those of ascorbic acid (VC) at all concentrations tested in this study. Nevertheless, the antioxidant activities of pinocembrin were improved by the complex with lecithin. There were significant differences in the antioxidation of the pinocembrin complex with lecithin among different antioxidant tests. At a concentration of 0.1 mg/mL, the hydroxyl radical scavenging effect of the pinocembrin–lecithin complex was nearly as good as that of VC, which were 69.96 ± 2.53% and 70.36 ± 0.92%, respectively; while it reached 82.44 ± 2.21%, only 17% lower than VC, and both were more effective than that of pinocembrin (72.83 ± 1.55%), at a concentration of 1.0 mg/mL. However, at 1.0 mg/mL, the ABTS radical scavenging ability of the pinocembrin–lecithin complex was just 24.73 ± 1.04%.

As illustrated in Figure 5, the antioxidant activity was proportional to the concentration of the pinocembrin complex with lecithin, the relationships between antioxidant activity and concentration were shown in Table 3. It appeared that the relations between the radical scavenging capacity for DPPH, ABTS, superoxide-anion, and the concentration of the pinocembrin–lecithin complex were significant linear functions; *R*^2^ (coefficient of determination) was 0.9266, 0.9906, and 0.9839, respectively. Furthermore, there was a conspicuous logarithmic function (*R*^2^ = 0.9236) between the radical scavenging capacity for hydroxyl and the concentration of the complex.

## 3. Discussion

Pinocembrin has a great potential medicinal value for the treatment of ischemic stroke, neurodegenerative and cardiovascular diseases [10,15]. However, its applications are seriously restricted by reason of low solubility. Lecithin possesses both a good hydrophilicity and lipophilicity and exhibits numerous bioactivities such as improvement in memory, liver protection, anti-aging, and regulation and control of blood glucose and lipids [28,29]. In the present study, the solubility analysis demonstrated that the water-solubility and lipid-solubility of pinocembrin complex with lecithin were much better than those of pinocembrin. The structural properties analysis implied that the pinocembrin–lecithin complex displayed the appearance of an amorphous state and no characteristic endothermic peak and, in contrast to the pinocembrin; while the spectra of pinocembrin–lecithin complex contained the characteristic absorption peaks of pinocembrin and lecithin, no novel absorption peak was found, suggesting that no novel covalent bond was produced in the pinocembrin and lecithin compound. These results are in agreement with the reports of other flavone complexes with lecithin [25,26].

Free radicals are molecules or fragments of molecules that contain an unpaired electron in their atomic or molecular orbitals, which include reactive oxygen species (ROS) and non-radical reactive oxygen species [30]. The most important ROS are the hydroxyl radical (^•^OH), superoxide-anion (O_2_^−^), hydrogen peroxide, and singlet oxygen, which can badly damage the membrane lipids, proteins, and DNA, leading to loss of function and eventually death of the organism [31,32]. In the current research, the results indicated that the radical scavenging activities of DPPH, ABTS, hydroxyl, and superoxide-anion of pinocembrin–lecithin complex were distinctly higher than those of pinocembrin. The scavenging capacities for hydroxyl radical, DPPH, superoxide-anion radical and ABTS radical pinocembrin–lecithin complex were 82.44 ± 2.21%, 40.07 ± 1.32%, 59.15 ± 0.86%, and 24.73 ± 1.04% at 1.0 mg/mL, respectively. This result is similar to previous studies [33,34]. It was suggested that the scavenging abilities of different free radicals were closely related to the structure of the pinocembrin–lecithin complex, which is identical to the report of Vargas-Sánchez et al. [35].

## 4. Experimental Section

### 4.1. Materials

**Figure 6:**
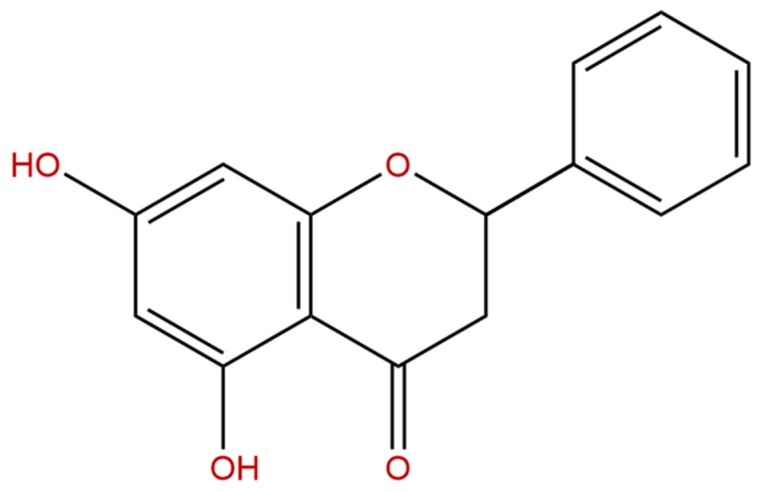
Chemical structure of pinocembrin.

The sample of pinocembrin (Figure 6) was offered from Xi’an Natural Field Bio-technique Co., Ltd. (Xi’an, China). The lecithin was purchased from Sigma-Aldrich (Shanghai, China) Trading Co., Ltd. (Shanghai, China). All other chemicals and reagents were analytically pure.

### 4.2. Preparation of Pinocembrin–Lecithin Complex

Pinocembrin (100 mg) and lecithin (200 mg) were solubilized in 50 mL of tetrahydrofuran and stirred for 4 h at 25 °C. Nitrogen purging was utilized to remove the tetrahydrofuran completely. The pinocembrin–lecithin complex was obtained after lyophilization [23].

### 4.3. Preparation of Physical Mixture of Pinocembrin and Lecithin

One hundred milligrams of pinocembrin and 200 mg of lecithin were uniformly mixed and stirred in a small beaker at 25 °C so that the physical mixture of pinocembrin and lecithin was produced.

### 4.4. Determination of Solubility in Water and n-octane

The solubility of pinocembrin in water and *n*-octane was determined following the previous method [36]. Briefly, 1.28 mg pinocembrin was weighed accurately and diluted with 5 mL methanol to attain the pinocembrin solution (1 mM). Pinocembrin solutions (0.1, 0.2, 0.4, 0.6, 0.8 mL) were diluted with methanol with 1 mL, and 1 mL distilled water or *n*-octane was added. Subsequently, the mixture was diluted with equal volumes of acetone, diethyl ether, and ethanol to 10 mL. One milliliter of methanol and 1 mL distilled water or *n*-octane were mixed and diluted with equal volumes of acetone, diethyl ether, and ethanol to 10 mL as a blank control. Finally, the absorbance was measured at 290 nm. The regression equations of solubility in water and *n*-octane were addressed as follows:

Y = 0.0001X + 0.0095 (*R*^2^ = 0.9934) (1)

Y = 7 × 10^−5^X + 0.0021 (*R*^2^ = 0.9969) (2)

where Y is the absorbance of pinocembrin, X is the concentration of pinocembrin. Two milligrams of pinocembrin and 5 mL distilled water or *n*-octane were incubated thoroughly for 8 h at room temperature. Then, the mixture was centrifuged at 4000 r/min for 10 min, and 1 mL supernate was collected, mixed with 1 mL methanol, and diluted with equal volumes of acetone, diethyl ether, and ethanol to 10 mL. Finally, the absorbance of pinocembrin was measured at 290 nm, and the apparent solubility in water and *n*-octane of pinocembrin was calculated by Equations (1) and (2), respectively. The apparent solubility in water and *n*-octane of the physical mixture and the complex was determined the same way.

### 4.5. Ultraviolet-Vis Spectroscopy

One milligram of pinocembrin, lecithin, their physical mixture, and the complex were dissolved in 10 mL of methanol, respectively. An UV-2500PC spectrophotometer (Shimadzu, Kyoto, Japan) was used to determine the UV-vis absorption spectra of pinocembrin, lecithin, their physical mixture, and the complex in the range from 230 to 400 nm.

### 4.6. Fourier-Transform Infrared Spectroscopy

One milligram of powdered samples (pinocembrin, lecithin, their physical mixture, and the complex) were mingled with dry potassium bromide (KBr) in proportion with 1:30 and crushed into a transparent tablet, respectively, and FTIR spectra analysis was performed on a VECTOR-22 infrared spectrophotometer (Bruker, Karlsruhe, Germany) over the wavenumber range from 4000 cm^−1^ to 400 cm^−1^.

### 4.7. Scanning Electron Microscopy

Layers of gold were dribbled onto pinocembrin, lecithin, their physical mixture, and the complex, and a SU1510 scanning electron microscope (Hitachi, Tokyo, Japan) was used to acquire the micrographs under 10 kV and in a low vacuum.

### 4.8. Differential Scanning Calorimetry

Thermograms of pinocembrin, lecithin, their physical mixture, and the complex were determined by a DSC60 (Shimadzu, Kyoto, Japan). The samples were sealed in an aluminum crimp cell and heated at the speed of 10 °C /min from 30 °C to 300 °C in nitrogen atmosphere. The in-built software (TA-60WS, Shimadzu, Japan) was made use of to record and process the data.

### 4.9. Determination of Antioxidant Activity In Vitro 

#### 4.9.1. 2,2′-Diphenyl-1-picrylhydrazyl Radical Scavenging Activity

2,2′-Diphenyl-1-picrylhydrazyl radical scavenging capacity was measured according to the reported method with some modifications [37]. Briefly, 0.2 mL of a freshly prepared DPPH ethanol solution (0.1 mM) was added into 0.2 mL of different concentrations (0.2, 0.5, 1.0, 1.5, and 2.0 mg/mL) samples. The mixtures were shaken equably and kept at indoor temperature in the dark for 30 min, and the absorbance was measured at 517 nm. The VC was served as a positive control. The radical elimination capacity of DPPH was computed by the following equation:

Scavenging ability (%) = [1 − (A_s_ − A_r_)/A_b_] × 100, where A_s_ is the absorbance of the sample, A_r_ is the absorbance of the sample except DPPH, and A_b_ is the absorbance of DPPH without the sample.

#### 4.9.2. 2,2′-Azino-bis(3-ethylbenzthiazoline-6-sulphonic acid) Radical Cation Scavenging Activity

The radical elimination capacity of ABTS was estimated following the previous method with a minor modification [38]. Briefly, equal volumes of ABTS (7.0 mM) and potassium persulfate solution (2.45 mM) were mixed, incubated for 12–16 h at room temperature, avoiding light, to generate a dark-colored solution containing ABTS radicals and then diluted with ethanol to an absorbance of 0.7 ± 0.02 at 734 nm. 2,2′-Azino-bis(3-ethylbenzthiazoline-6-sulphonic acid) (ABTS) solution (3.9 mL) was added into 0.1 mL of different samples of varying concentrations (4.0, 10.0, 20.0, 30.0, and 40.0 mg/mL) and left to react for 30 min in the dark. The absorbance was measured at 734 nm against a blank (ethanol). The VC was used as a positive control. The percentage reduction of ABTS radical cation was calculated as follows:

Scavenging ability (%) = [1 − A_s_/A_b_] × 100, where A_b_ is the absorbance of ABTS with ethanol and A_s_ is the absorbance of the sample and ABTS.

#### 4.9.3. Hydroxyl Radical Scavenging Activity

The scavenging activity of samples for hydroxyl radical was detected using the described method [39] with minor modifications. Briefly, each sample solution (0.4, 1.0, 2.0, 3.0, and 4.0 mg/mL, 2.0 mL) was mixed thoroughly with FeSO_4_ (6.0 mmol/L, 2.0 mL) and H_2_O_2_ (6.0 mmol/L, 2.0 mL) and incubated for 10 min. Then, salicylic acid (6.0 mmol/L, 2.0 mL) was added into the mixture that was kept for 30 min in a thermostatic water bath at 37 °C. Finally, the absorbance was measured at 510 nm. The VC served as a positive control. The activity of each sample to scavenge hydroxyl radicals was evaluated by the following formula:

Scavenging ability (%) = [1 − (A_s_ − A_r_)/A_b_] × 100, where A_s_ is the absorbance of the sample, A_r_ is the absorbance of the control (distilled water instead of salicylic acid), and A_b_ is the absorbance of the blank (distilled water instead of sample).

#### 4.9.4. Superoxide-Anion Radical Scavenging Activity

The radical elimination capacity of samples for superoxide-anion was determined by the reported method [40]. The Tris-HCl solution (0.05 M, pH 8.2, 4.5 mL) was incubated for 20 min in a thermostatic water bath at 25 °C. Then, 0.4 mL of 1,2,3-phentriol (25 mM) and 1.0 mL of each sample solution (0.69, 1.725, 3.45, 5.175, and 6.90 mg/mL) were added into the reaction mixture in turn followed by an incubation for 5 min in the thermostatic water bath at 25 °C. Finally, the reaction was terminated by the addition of a hydrochloride solution (HCl, 8 mM, 1.0 mL) and the absorbance was measured at 299 nm. The VC was used as a positive control. The percentage scavenging of superoxide-anion radical was estimated with the equation as follows:

Scavenging ability (%) = [1 − A_s_/A_b_] × 100, where A_s_ is the absorbance of the reaction mixture with the sample and A_b_ is the absorbance of the reaction mixture without the sample replaced by distilled water.

### 4.10. Statistical Analysis

All experiments were repeated in triplicate and data were analyzed as mean ± standard deviation (SD) with SPSS 25.0. One-way analysis of variance (ANOVA, IBM Corporation, Beijin, China) was utilized to assess the differences between the means.

## 5. Conclusions

In this research, a pinocembrin–lecithin complex was successfully prepared, and its physicochemical properties were analyzed by UV, FTIR, SEM, DSC, and solubility assay. The pinocembrin–lecithin complex significantly improved the solubility of pinocembrin in water and *n*-octane. The antioxidant activities of the pinocembrin–lecithin complex were evaluated via radical scavenging capacities for DPPH, ABTS, hydroxyl, and superoxide-anion. It was demonstrated that the antioxidant activities of pinocembrin were obviously enhanced by the complex with lecithin. It suggested that pinocembrin–lecithin complex has a great potential as an application prospect in the healthcare industry and clinical setting.

## Figures and Tables

**Table 1:** Characterization methods of pinocembrin–lecithin complex.

No.	Characterization Methods
1	Ultraviolet spectrum (UV)
2	Fourier transform infrared spectroscopy (FTIR)
3	Scanning electron microscope (SEM)
4	Differential scanning calorimetry (DSC)

**Table 2:** Apparent solubility of pinocembrin, its physical mixture with lecithin and the complex in water and *n*-octane.

Solvent	Apparent Solubility (μg/mL)		
	Pinocembrin	Physical Mixture	Complex
Water	48.33	178.33	265.00
*n*-Octane	65.24	84.29	165.24

**Table 3:** The relationship between antioxidant activity and concentration of pinocembrin–lecithin complex.

Antioxidant Test	Best Fitting Function	Determination Coefficient (*R*^2^)
DPPH	Y = 0.0264X + 0.2801	0.9266
ABTS	Y = 0.0417X + 0.0440	0.9906
Hydroxyl	Y = 0.0754Ln(X) + 0.7120	0.9236
Superoxide-anion	Y = 0.0379X + 0.4076	0.9839

X was the concentration of the pinocembrin–lecithin complex, Y was the antioxidant activity corresponding to X.
